# Reflections on working as and supervising trial therapists on trials of psychosocial interventions

**DOI:** 10.1192/bjo.2024.709

**Published:** 2024-07-25

**Authors:** Katherine Berry, Joanne Ellis, Daniel Pratt, Gillian Haddock

**Affiliations:** Division of Psychology and Mental Health, School of Health Sciences, University of Manchester, UK; and Department of Research and Innovation, Greater Manchester Mental Health NHS Foundation Trust, Manchester, UK; Division of Nursing Midwifery and Social Work, University of Manchester, UK; and Pennine Care NHS Foundation Trust, Manchester, UK

## Abstract

This editorial provides an overview of the challenges and benefits of working as and supervising trial therapists from the perspective of investigators and trial therapists. Key differences between trial therapy and standard care are considered, with recommendations for best practice.

The UK government vision is for research to be integrated throughout healthcare services.^1^ Accordingly, National Health Service NHS Trusts, which are the UK government-funded organisations that administer patient care, all host departments of research and innovation which in theory are committed to delivering research to improve clinical practice. New treatments should be evaluated through randomised controlled trials and these are essential for driving healthcare innovation.^2,3^ Trials in the UK are typically funded by the National Institute of Health Research or UK Research and Innovation. These funders cover the costs of researchers to carry out the evaluation of the intervention. However, to ensure ongoing commitment beyond the duration of the trial, funders do not cover additional costs associated with delivering the new intervention (so-called excess treatment costs (ETCs)). These costs need to be met by NHS Trusts. Once funding is obtained, there are a number of challenges to the successful delivery of trials.

First and foremost, it can be difficult to find people to deliver the interventions as part of the trial. There may be a lack of available staff with the appropriate level of professional training and expertise. For example, many trusts currently have staff shortages, meaning that there are not enough staff to deliver routine care, let alone interventions as part of a research trial. Relatedly, clinicians, typically nurses or allied health professionals, often report lacking in knowledge or experience of research processes and do not see it as part of their role.^4^ Second, trusts may be reluctant to fund trial therapy posts. Trusts can apply to service commissioners to cover ETCs. However, each trust has a threshold for claiming ETCs which means they need to absorb costs below the threshold, and the payment of ETCs is dependent upon meeting recruitment targets, so trusts need to be confident the trial can recruit to target.

A further issue related to both the payment of the intervention delivery and identification of people to deliver the intervention is that research studies are funded on a fixed-term basis over relatively short lengths of time. This means that intervention deliverers are employed on short, fixed-term contracts which are undesirable for staff and risk expertise being lost from the trust when the trial ends. To circumvent issues presented by fixed-term contracts, secondment opportunities can be offered to people in existing posts, but when resources are tight, managers are unable to release people from their roles in this way. Trusts may also ask people in existing posts to deliver interventions as part of their existing role or to pick up extra hours, but it may be difficult to ensure protected time is made available to deliver the trial therapy. Alternatively, trusts may employ people as trial therapists, with these individuals moving between studies when needed or providing a critical mass who can work across different studies at the same time. However, this scenario relies on trusts having a portfolio of studies with a succession of funded research.

Once intervention delivers are in post, the role itself can present challenges. First, there may be additional work for therapists, compared with routine care. In trials it is important to document intervention processes for the purposes of assessing fidelity to a specific model. These assessments help the understanding of trial outcomes. For example, if there are no differences between the new treatment and usual care, it is important to know that the new therapy was delivered to the standard expected. Although important for the research, an additional assessment process can create a burden for staff. Relatedly, trial therapists may experience extra scrutiny of their work which adds a sense of pressure to deliver an intervention to a high standard. The need to audio-record intervention sessions is a good example of a routine procedure to assess fidelity in trials, which can raise therapists’ anxieties. However, increased burden and therapist anxieties can be balanced against the benefits of additional training and more frequent supervision than typically received in routine practice. The cost for this training and supervision is typically funded by the research grant and can also be an excellent opportunity for personal and professional development.

Second, trial therapists have limited flexibility to deliver interventions according to their own beliefs and ways of practising. Although trials vary in how much flexibility is permitted in intervention delivery, it is important to ensure that everyone in the treatment arm of the trial receives the essential components of the intervention, and that delivery remains aligned to the core principles of the intervention. This issue can lead to particular tensions when the participant may meet inclusion criteria for the study but may not want or be ready to work on the problem area that is the focus of the intervention being trialled, perhaps because other issues are more pressing. In routine practice, a therapist might wait to commence an intervention until the client is ready, but these decisions are more pressured within trials because of trial funding timelines. Similarly, trial therapists are often more constrained than in routine care in terms of the number of sessions that can be offered and the time window within which sessions need to happen, because of the need to complete therapy before follow-up assessments are due.

A third challenge for therapists is isolation from clinical teams. Many trial therapists are managed within the research departments of employing trusts, rather than within the routine care teams. To prevent the effects of trial therapists’ expertise in the new intervention reaching individuals not receiving the new intervention in the control arm of the trial, trial therapists may have to work ‘at a distance’ from the routine care team (although the sharing of risk-pertinent information would always be expected between the trial therapy team and the routine care team). Managing separation of roles can be particularly difficult when people occupy dual roles as trial therapists and clinicians within the service, and can generate additional dilemmas, for example the issue of whether to share knowledge generated for research purposes with the wider care team.

Despite these issues with identifying and employing trial therapists and the challenges the role may present for therapists themselves, we argue that there are many benefits to delivering trial therapy at an organisational and individual level. For example, there is evidence that research-active trusts have better patient outcomes^5^ as well as staff recruitment and retention.^6^ From an organisational perspective, training the workforce in new treatments also helps ensure that new therapies (if demonstrated to be effective) will be more likely to be available for service users following the end of the study. As highlighted previously, therapist training is typically covered by the research funders rather than trusts themselves, and resources can also be disseminated to other clinicians via supervision or workshops offered by trial therapists. Access to evidenced-based psychological interventions in routine care remains limited, and therapy trials which do not operate waiting lists therefore enable service users to access psychological interventions with experienced therapists in a more timely manner. From a therapist's perspective, working as a trial therapist not only helps staff to keep their own research skills active but also affords the opportunity to be part of a process that generates evidence-based practice.

Between us, we have experience of successfully delivering multiple trials of complex intervention in the NHS which have been influential in informing clinical guidance and health service practice. This includes but is not limited to trials of cognitive–behavioural therapies for psychosis and other severe mental health problems, and trials in a range of different settings including in-patient wards and prisons (e.g.^7–9^). On the basis of these experiences, we would recommend working with healthcare providers to explore the range of different ways in which therapists can be employed and to use these different means flexibly depending on the local context. Researchers also need to build good relationships with clinical services to identify ways of developing local research capacity (for example, incorporating research into job roles and appraisals). We further recommend using recruitment drives for therapists which sell the benefits of involvement in research, and also supportive training and supervision structures which bring research therapists together to share experiences. On a national level, it is also important to empirically demonstrate the added value of research activity for individual clinicians, patients and the healthcare organisation as a whole. See Table 1 for a summary of the ideas discussed in this paper and our recommendations for best practice.

**Table 1:** Summary of challenges and benefits to delivering trial therapy and recommendations for best practice

Challenge	Benefit to individuals and organisations
*Additional costs* The additional costs of delivering new interventions are not covered by funding bodies. Local commissioning arrangements need to be in place to meet these excess treatment costs. Such costs are only covered by Local Commissioning Groups if trusts have reached a pre-set threshold and payment is dependent on recruitment targets being met.	*Specialist training* Trial therapists are trained in the new intervention, which enables staff to enhance or develop additional therapeutic techniques and competencies.
*Recruitment of therapists* It can be difficult to recruit staff to deliver the intervention. For example, staff may not feel they have sufficient research skills or experience; the short-term nature of trials means post are funded on a fixed-term basis; service pressures can prevent staff from being able to take up secondment opportunities and trial therapist posts; and substantive posts may not be viable for less research-active organisations.	*Specialist supervision* Trial therapists are supervised more often than in routine care. This is typically provided by international experts in the field and may also include peer supervision where trial therapists can share ideas.
*Additional paperwork for therapists* Trial therapists are often required to complete additional documentation to enable the assessment of therapy processes.	*Involvement in innovations and the generation of evidence-based care* Staff and organisations can contribute to the development of innovative approaches which may improve outcomes. If the intervention is shown to be effective, trained staff can disseminate skills and embed the new treatment into routine care.
*Additional scrutiny for therapists* The use of audio or video recordings to assess therapist competence or adherence to the intervention being tested can raise staff anxieties.	*Improving access to psychological therapies* Trial therapy is in addition to what is offered in routine care, increasing access to psychological therapy with highly trained therapists. Participants typically commence treatment much sooner than in routine care, where long waiting times for psychological therapy are commonplace.
*Constraints of delivering trial therapy* Therapy may need to focus on one specific problem area, be over a set number of sessions and be delivered within a set window of time.	*Patient outcomes* Access to innovative psychological interventions (if demonstrated to be effective) can decrease symptom severity and distress, improve functioning and enhance recovery.
*Therapist isolation from routine care team* To prevent contamination of usual care, trial therapists may need to sit outside of the routine care team which can present dilemmas in terms of the level of information sharing, and challenges in terms of therapists feeling separate from the clinical team.	*Staff recruitment and retention* Research-active trusts demonstrate improved staff recruitment, retention and reputational benefits.
*Recommendations*	
Trusts need to promote a research culture by ensuring that research is an integral part of staff job roles and thus part of appraisal processes.	
Trusts can support staff to take up trial therapy opportunities by using a flexible approach to recruitment including secondment opportunities (with backfill), the offer of additional hours, and dedicated trial therapist posts.	
Trusts need to ensure that staff delivering trial therapy alongside other roles need to be given ring-fenced time to engage in training, supervision and delivery of therapy.	
Researchers need to promote the additional benefits of delivering trial therapy to staff to offset concerns about additional burden and scrutiny (e.g. additional training and supervision from experts in the field).	
Researchers need to ensure that the collection of trial therapy processes measures is built into the therapist's time, and that it is supported by research staff wherever possible and kept to a minimum.	
Researchers need to be sensitive to the potential of therapist anxiety about the additional scrutiny, and specifically work with therapists to address this anxiety.	
Researchers need to promote to managers the additional benefits of delivering trial therapy, to offset concerns about additional strains the trial may place on service delivery (e.g. it may ease waiting lists and upskill workforce with no extra training costs).	
Researchers need to ensure that trial therapists receive adequate peer support in their roles, to offset isolation they may experience from the routine care team and offer supervision around dilemmas in the sharing of research information with clinical teams.	

## Data Availability

Data availability is not applicable to this article as no new data were created or analysed in this study.
